# Well-defined survival outcome disparity across age cutoffs at 45 and 60 for medullary thyroid carcinoma: a long-term retrospective cohort study of 3601 patients

**DOI:** 10.3389/fendo.2024.1393904

**Published:** 2024-06-14

**Authors:** Kun Zhang, Xinyi Wang, Tao Wei, Zhihui Li, Jingqiang Zhu, Ya-Wen Chen

**Affiliations:** ^1^ Division of Thyroid Surgery, Department of General Surgery, West China Hospital, Sichuan University, Chengdu, Sichuan, China; ^2^ Department of Otolaryngology, Icahn School of Medicine at Mount Sinai, New York, NY, United States; ^3^ Department of Cell, Developmental and Regenerative Biology, Icahn School of Medicine at Mount Sinai, New York, NY, United States; ^4^ Black Family Stem Cell Institute, Icahn School of Medicine at Mount Sinai, New York, NY, United States; ^5^ Institute for Airway Sciences, Icahn School of Medicine at Mount Sinai, New York, NY, United States; ^6^ Center for Epithelial and Airway Biology and Regeneration, Icahn School of Medicine at Mount Sinai, New York, NY, United States

**Keywords:** medullary thyroid cancer, survival analysis, age cutoffs, well-defined survival disparity, outcome predictor

## Abstract

**Background:**

Medullary thyroid cancer (MTC) is a challenging malignancy. The survival outcome of MTC based on AJCC staging system does not render a discriminant classifier among early stages.

**Methods:**

3601 MTC patients from 2000 to 2018 were identified from the Surveillance, Epidemiology, and End Results (SEER) database. Smooth curve fitting, Cox proportional hazard regression and competing risk analysis were applied.

**Results:**

A linear correlation between age and log RR (relative risk of overall death) was detected. Overlaps were observed between K-M curves representing patients aged 45–50, 50–55, and 55–60. The study cohort was divided into 3 subgroups with 2 age cutoffs set at 45 and 60. Each further advanced age cutoff population resulted in a roughly “5%” increase in MTC-specific death risks and an approximately “3 times” increase in non-MTC-specific death risks.

**Conclusions:**

The survival outcome disparity across age cutoffs at 45 and 60 for MTC has been well defined.

## Introduction

Medullary thyroid cancer (MTC), whether inherited or sporadic, is an uncommon and challenging malignancy (1, 2). Since calcitonin (Ct) secreting parafollicular C cells of the thyroid gland were first publicly described by a pathologist (3) to be the origin of MTC, many efforts (4–7) in oncological study and clinical epidemiology were made to demonstrate that up to 25% of all cases are autosomal dominant inherited disorders correlating to RET gene alterations, with about 75% presenting as sporadic tumors. Within less than a decade of these findings, it was discovered that almost all patients with multiple endocrine neoplasia types 2A and 2B (MEN2A and MEN2B) as well as familial medullary thyroid cancer (FMTC) had RET germline mutations, while approximately 50 percent of sporadic MTCs had somatic RET mutations (8–13). Thus, MTC has been well recognized as a proto-oncogene driven disease.

According to current Surveillance, Epidemiology, and End Results (SEER) data, in the United States, MTC has accounted for 2% to 3% of new diagnoses of all thyroid cancers each year for the last three decades (14). Unlike the rising incidence rates of papillary thyroid cancer (PTC) and other differentiated thyroid cancers (DTC), incidence rates for MTC have remained relatively stable over the same period of time (15). A subsequent analysis of MTC patients using the SEER database demonstrated that the 7th and 8th editions of the AJCC (The American Joint Committee on Cancer) staging system were associated with five-year overall survival (OS) rates of 95% in stage I, 91% in stage II, 89% in stage III, and 68% in stage IV. Furthermore, cancer-specific survival (CSS) rates were 100% in stage I, 99% in stage II, 97% in stage III, and 82% in stage IV (16). The survival outcome, whether OS or CSS, of MTC based on the current AJCC staging system did not render a discriminant classifier with good disparity validity among early stages (stage I, II and III). One explanation might be that there are key survival factors, other than staging itself, that can predict survival outcomes in a more discriminant manner. Age may be one of the key survival predictors for MTC. On one hand, studies (17–19) have shown that patient age at diagnosis is an independent prognostic factor. On the other hand, borrowing from the 2015 ATA (American thyroid association) guideline on DTC management (20), an age cutoff of 55 as a staging threshold has been well established. With this in mind, the impact of age on survival prognosis of MTC may be profound, and has not been thoroughly investigated.

MTC’s low incidence and unbalanced staging-prognosis method has impeded the study of prognostic outcomes, as well as the refinement of the cancer risk stratification and staging system. To fill this knowledge gap, we sought to extract data from the SEER database to acquire a large MTC cohort to evaluate the impact of age on survival outcomes and other associated factors of MTC patients with high reliability and rigorousness.

## Materials and methods

### Data sources

Our study utilized a cohort of pathologically confirmed MTC patients from the SEER program, a registry that recodes cancer incidence and mortality data from 18 population-based cancer registries across the United States, which comprises approximately 27.8% of the U.S. population (21). As SEER is an open-access public database, no institutional review board approval was required. SEER registries collect data concerning patient demographics, tumor morphology, stage at diagnosis, primary tumor site, the first course of treatment, and follow-up for vital status and causes of deaths. Our selected database is cited as: “Incidence - SEER Research Plus Data, 18 Registries, Nov 2020 Sub (2000–2018) - Linked To County Attributes - Total U.S., 1969–2019 Counties, National Cancer Institute, DCCPS, Surveillance Research Program, released April 2021, based on the November 2020 submission.”

### Study population

Using the selected database, we identified a consecutive cohort of 3601 patients diagnosed with MTC between 2000 and 2018. The clinical data were extracted regarding demographics, tumor staging, and therapeutic approaches: age, gender, race, primary site of tumor, pathology, AJCC (American Joint Committee on Cancer) stage, SEER stage [defined by the SEER database referred to as the SEER stage in our article that represents the staging schema based on information about primary site, histology, or other factors (22)], primary surgery, lymph node dissection, external beam radiation therapy (EBRT), chemotherapy, systemic therapy, survival months, cause-specific death classification, and survival status.

### Outcome definition

Cause of death was defined to record whether each patient died as a result of MTC or any causes other than MTC, or whether they were alive at the end of the follow-up period. In our study, the primary outcome was OS, which was defined as the time between initial diagnosis and death from all causes. As the secondary outcome, OS was split as CSS and deaths from causes (OC) other than MTC, and analyzed with competing risks regression (23). The focus on OC or non-MTC-specific mortality is justified because OC represents a mutually exclusive survival event, serving as the counterpart of CSS. This distinction allows for the use of competing risk regression, which aims to analyze the specific contributions of different types of mortality to OS.

### Smooth curve fitting between age and overall relative death risk

To examine the association pattern between age and OS of MTC patients, we applied a generalized additive model (GAM) to conduct smooth curve fitting and to examine whether the impact of age on overall death risk is linear or partitioned into intervals. This allowed us to determine whether the relationship with OS started to drastically change when patients reached a certain age. According to the AJCC 7th Edition/TNM Classification system, it is recommended that the population diagnosed with DTC at the age of 45 and older (including age 45) should be applied to a distinct staging algorithm. To maintain consistency with this globally accepted staging methodology, we include patients diagnosed at the ages of 50 and 55 in the age cutoff groups of 50–55 and 55–60, respectively.

### Survival analysis across age cutoffs

To determine the optimal age interval for defining the most discriminant survival disparity, Kaplan-Meier (K-M) curves were estimated and compared between two different age cutoffs. Associated prognostic factors were then selected by univariate Cox proportional hazards regression and K-M curves. Subsequently, significant factors screened by the univariate Cox regression (P < 0.05) were further included in multivariate Cox proportional hazards models. The best Cox model was selected by backward selection process (entry criterion: P < 0.05, elimination criterion: P > 0.10). Multivariate Cox proportional hazards regression analysis was performed to identify variables that independently affected the OS for MTC patients.

### Competing risk analysis between MTC-specific death and death from OC

In the competing risk model, deaths from OC were regarded as a competing event for MTC-specific death. We computed the cumulative incidence function (CIF) for MTC and OC. CIF curves of MTC-specific death and OC across age cutoff groups were plotted and tested. Subgroup analysis was subsequently carried out according to age, sex, AJCC TNM, tumor size, tumor extension, primary surgery, lymph node dissection, EBRT, chemotherapy, and systemic therapy. The significance in CIF values among subgroups were evaluated by Fine & Gray’s test (23). In the end, we calculated the sub-distribution hazard ratio (SHR) of the included variables for MTC patients based on the multivariate competing risk model with the R package “riskRegression”.

### Statistical analysis

We presented descriptive statistics in **Table 1** for the entire study cohort and compared the results across age cutoffs at 45 and 60. Continuous and categorical variables were assessed with the Kruskal-Wallis test and Pearson chi-square test, respectively. Continuous variables were expressed as the mean ± standard deviation (SD)/median. Categorical variables were shown as numbers (percentages). All statistical analyses were carried out employing the R studio version 4.0.4. A two-tailed P < 0.05 was considered statistically significant.

**Table 1:** Demographic and clinical characteristics by age cutoff groups in medullary thyroid cancer patients.

		Age cutoff			
	Total	<=45	>45 and <=60	>60	P-value
**N**	3601	1040	1171	1390	
**Age, Mean ± SD**	53.9 ± 17.2	32.5 ± 10.6	53.1 ± 4.3	70.7 ± 6.9	<0.001
**Sex, N (%)**					
Female	2088 (58.0%)	633 (60.9%)	702 (59.9%)	753 (54.2%)	
Male	1513 (42.0%)	407 (39.1%)	469 (40.1%)	637 (45.8%)	
**Race, N (%)**					<0.001
White	3016 (83.8%)	832 (80.0%)	969 (82.7%)	1215 (87.4%)	
Black	317 (8.8%)	115 (11.1%)	112 (9.6%)	90 (6.5%)	
^a^Otders	268 (7.4%)	93 (8.9%)	90 (7.7%)	85 (6.1%)	
**Patdological Grade, N (%)**					0.003
Well	90 (2.5%)	26 (2.5%)	27 (2.3%)	37 (2.7%)	
Moderate	85 (2.4%)	17 (1.6%)	35 (3.0%)	33 (2.4%)	
Poor	95 (2.6%)	11 (1.1%)	32 (2.7%)	52 (3.7%)	
Undifferentiated	25 (0.7%)	5 (0.5%)	8 (0.7%)	12 (0.9%)	
Unspecified	3306 (91.8%)	981 (94.3%)	1069 (91.3%)	1256 (90.4%)	
**SEER stage, N (%)**					<0.001
Localized	1504 (41.8%)	419 (40.3%)	501 (42.8%)	584 (42.0%)	
Regional	917 (25.5%)	271 (26.1%)	314 (26.8%)	332 (23.9%)	
Distant	353 (9.8%)	70 (6.7%)	116 (9.9%)	167 (12.0%)	
Unstaged	827 (23.0%)	280 (26.9%)	240 (20.5%)	307 (22.1%)	
**AJCC T, N (%)**					0.061
T1	562 (15.6%)	162 (15.6%)	181 (15.5%)	219 (15.8%)	
T2	290 (8.1%)	70 (6.7%)	113 (9.6%)	107 (7.7%)	
T3	263 (7.3%)	62 (6.0%)	85 (7.3%)	116 (8.3%)	
T4	101 (2.8%)	24 (2.3%)	38 (3.2%)	39 (2.8%)	
Unspecified	2385 (66.2%)	722 (69.4%)	754 (64.4%)	909 (65.4%)	
**AJCC N, N (%)**					0.160
N0	1312 (36.4%)	364 (35.0%)	437 (37.3%)	511 (36.8%)	
N1a	366 (10.2%)	106 (10.2%)	131 (11.2%)	129 (9.3%)	
N1b	488 (13.6%)	141 (13.6%)	172 (14.7%)	175 (12.6%)	
Unspecified	1435 (39.9%)	429 (41.2%)	431 (36.8%)	575 (41.4%)	
**AJCC M, N (%)**					0.003
M0	1182 (32.8%)	314 (30.2%)	404 (34.5%)	464 (33.4%)	
M1	127 (3.5%)	22 (2.1%)	44 (3.8%)	61 (4.4%)	
Unspecified	2292 (63.6%)	704 (67.7%)	723 (61.7%)	865 (62.2%)	
**Tumor size, N (%)**					<0.001
<=1cm	551 (15.3%)	192 (18.5%)	187 (16.0%)	172 (12.4%)	
>1cm and <=2cm	542 (15.1%)	132 (12.7%)	185 (15.8%)	225 (16.2%)	
>2cm and <=3cm	430 (11.9%)	128 (12.3%)	157 (13.4%)	145 (10.4%)	
>3cm and <=4cm	241 (6.7%)	75 (7.2%)	89 (7.6%)	77 (5.5%)	
>4cm and <=5cm	163 (4.5%)	38 (3.7%)	49 (4.2%)	76 (5.5%)	
>5cm	223 (6.2%)	50 (4.8%)	71 (6.1%)	102 (7.3%)	
Unspecified	1451 (40.3%)	425 (40.9%)	433 (37.0%)	593 (42.7%)	
**Tumor extension, N (%)**					0.019
Confined to tdyroid capsule	1797 (49.9%)	518 (49.8%)	604 (51.6%)	675 (48.6%)	
T3b	227 (6.3%)	64 (6.2%)	89 (7.6%)	74 (5.3%)	
T4a	126 (3.5%)	32 (3.1%)	46 (3.9%)	48 (3.5%)	
T4b	68 (1.9%)	13 (1.2%)	19 (1.6%)	36 (2.6%)	
Unspecified	1383 (38.4%)	413 (39.7%)	413 (35.3%)	557 (40.1%)	
**Surgery, N (%)**					<0.001
No surgery	326 (9.1%)	60 (5.8%)	89 (7.6%)	177 (12.7%)	
Lobectomy	167 (4.6%)	30 (2.9%)	56 (4.8%)	81 (5.8%)	
Total tdyroidectomy	2966 (82.4%)	913 (87.8%)	988 (84.4%)	1065 (76.6%)	
Otder surgery	142 (3.9%)	37 (3.6%)	38 (3.2%)	67 (4.8%)	
**Neck dissection, N (%)**					<0.001
No	939 (26.1%)	207 (19.9%)	281 (24.0%)	451 (32.4%)	
Yes	2224 (61.8%)	676 (65.0%)	760 (64.9%)	788 (56.7%)	
Unspecified	438 (12.2%)	157 (15.1%)	130 (11.1%)	151 (10.9%)	
**EBRT, N (%)**					0.124
No/Unknown	3226 (89.6%)	946 (91.0%)	1051 (89.8%)	1229 (88.4%)	
Yes	375 (10.4%)	94 (9.0%)	120 (10.2%)	161 (11.6%)	
**Chemotderapy, N (%)**					0.255
No/Unknown	3397 (94.3%)	991 (95.3%)	1103 (94.2%)	1303 (93.7%)	
Yes	204 (5.7%)	49 (4.7%)	68 (5.8%)	87 (6.3%)	
**Systemic tderapy, N (%)**					0.036
No/Unknown	2230 (61.9%)	631 (60.7%)	702 (59.9%)	897 (64.5%)	
Yes	1371 (38.1%)	409 (39.3%)	469 (40.1%)	493 (35.5%)	
**Cause of deatds, N (%)**					<0.001
Censored	2689 (74.7%)	915 (88.0%)	924 (78.9%)	850 (61.2%)	
MTC	462 (12.8%)	86 (8.3%)	148 (12.6%)	228 (16.4%)	
Otder causes	450 (12.5%)	39 (3.8%)	99 (8.5%)	312 (22.4%)	
**Survival montds, Mean ± SD/Median**	78.4 ± 61.5/65	94.3 ± 65.0/91	83.1 ± 62.2/74	62.7 ± 54.1/47	0.001

a Others, American Indian/Alaska Native, Asian/Pacific Islander; SEER, the Surveillance, Epidemiology, and End Results program; SEER stage: see materials and methods; AJCC, American joint committee on cancer; EBRT, external beam radiation therapy; MTC, medullary thyroid carcinoma.

## Results

Using the SEER database from 2000–2018, 3612 consecutive MTC patients were identified, of which 11 were excluded from the study cohort due to no record of survival time. In total, 3601 MTC patients were analyzed. Epidemiologically, MTC patients can be characterized as a group of people with the following features: middle-aged, (median age 53.9 years old, range from 36.7 to 71.1) mostly white (3016, 83.8%), and slightly female predominant (male to female sex ratio is approximately 4 to 6). The median follow-up of the study cohort was 65 months (range from 1 month to 227 months). The total number of deaths during the follow-up period was 912 (25.3%), of which 462 (12.8%) were attributed to MTC and 450 (12.5%) to non-MTC causes rendering a balanced cause-of-death attribution. Overall, the 5- and 10-year CSS was 89.0% (CI: 87.9%–90.2%) and 82.8% (CI: 81.2%–84.4%), respectively, while the 5- and 10-year OS was 80.9% (CI: 79.5%–82.4%) and 68.5% (CI: 66.6%–70.5%).

According to the SEER combined staging system, about 40% of MTC patients (1504, 41.8%) were classified as local stage, about 25% (917, 25.5%) as regional stage, and about 10% (353, 9.8%) as distant stage. AJCC TNM staging method (24) was adopted by the SEER database. However, because unspecified data (T stage, 2385, 66.2%; M stage, 2292, 63.6%) made up around 65% of the total, AJCC staging was not included the following statistical analysis. Almost half of MTC foci were located within the thyroid capsule, and no less than 10% (T3b, T4a, and T4b combined: 421, 11.7%) of them extended beyond the thyroid capsule. Likelihood of lymph node metastasis for the MTC cohort was 23.8% (10.2% central neck compartment involved, 13.6% lateral neck compartment involved, and 39.9% lymph node involvement unspecified). With regard to the treatment regimen, the vast majority of MTC patients were treated with total thyroidectomy (82.4%), while a small fraction underwent lobectomy (4.6%), while neck dissection was performed in more than 60% of all cases (2224, 61.8%). Among the MTC cohort, 3 age cutoff groups were created based on our analysis, which will be described in detail in the next paragraph. All demographic data and baseline clinical characteristics across the age cutoffs at 45 and 60 are shown and compared in **Table 1**.

**Figure 1:**
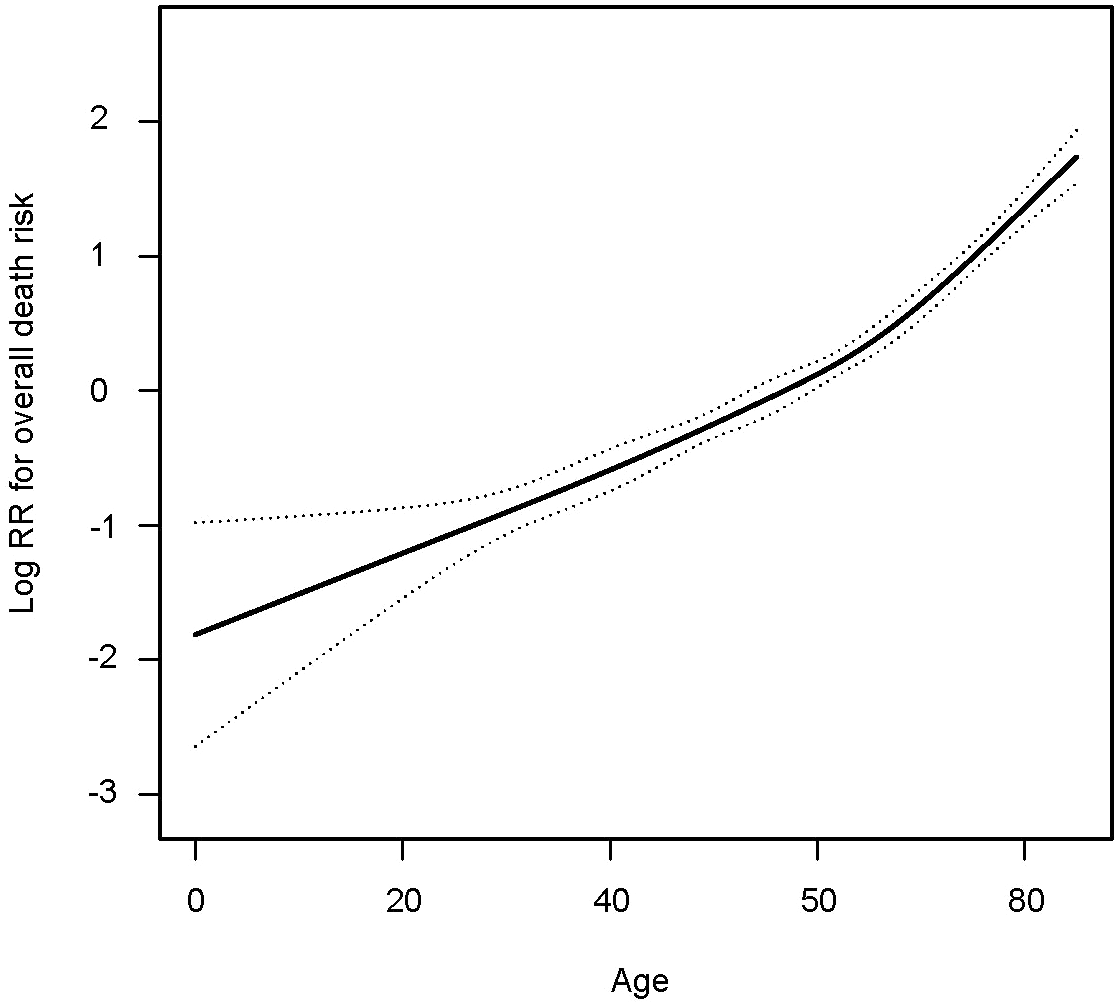
Smooth curve fitting showing the association between age in years and relative risk of overall death in patients with medullary thyroid cancer.

**Figure 2:**
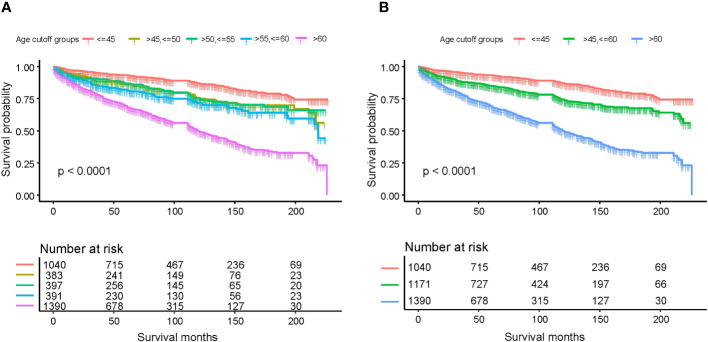
Kaplan-Meier survival curves estimating various age subgroups for overall survival based on different age cutoffs: **(A)** age cutoffs set at 45, 50, 55, and 60; **(B)** age cutoffs set at 45 and 60.

To determine the impact of age on overall survival probability, we first applied a generalized additive model (GAM) to conduct smooth curve fitting to examine whether the correlation between age and relative risk of overall death is linear or whether there is a threshold effect. The smooth curve fitting plotted in **Figure 1** illustrates that the association between age at diagnosis and overall relative risk of death (Log RR) was linear (Likelihood ratio test, P<0.001; Wald test, P<0.001; log rank test, P<0.001). Based on this discovery, we speculated that the cumulative risk of death should be smoothly partitioned into intervals corresponding with age cutoff groups. In order to demonstrate this, we further plotted five K-M survival curves (**Figure 2A**) that represent age groups with cutoffs set at 45, 50, 55, and 60, respectively. Overlaps were observed among K-M curves representing patients’ ages of 45–50, 50–55, and 55–60, indicating that survival differences at these age intervals are not statistically significant. In other words, a merger of all age groups from 45 to 60 is statistically justifiable. This was further supported by an additional set of K-M curves with evident survival disparities shown in **Figure 2B** (p<0.001). We, therefore, stratified the study cohort into 3 age groups (<45, >=45 and <60, and >60) for subsequent analyses.

In the multivariate Cox analysis for OS, advanced categorical age cutoff group (e.g., age>45 and <=60 vs. age<=45, HR=1.98, 95% CI 1.59–2.45, P<0.001) was estimated as the leading independent prognostic factors associated with worse overall survival outcomes, followed by higher AJCC M stage (M1 vs. M0, HR=1.98, 95% CI 1.48–2.65, P<0.001), more invasive tumor extension (T3a vs. confined to thyroid capsule, HR=2.23, 95% CI 1.67–2.98, P<0.001), larger tumor size (e.g., >3cm and <=4cm vs. <= 1cm, HR=1.77, 95% CI 1.23–2.53, P=0.002), advanced AJCC N stage (N1b vs N0, HR=1.36, 95% CI 1.07–1.73, P=0.013), black ethnicity (black people vs. white people, HR=1.37, 95% CI 1.09–1.73, P=0.008), and male gender (male vs. female, HR=1.27, 95% CI 1.11–1.46, P=0.001) after adjustment and model selection from the univariate Cox regression. Surgery (e.g., total thyroidectomy vs. no surgery, HR=0.25, 95% CI 0.20–0.30, P<0.001) and systemic therapy (yes vs. no, HR=0.75, 95% CI 0.62–0.90, P=0.002) were estimated to be the only independent predictors for better OS. Neck dissection and AJCC T stage were not relevant to OS. Although EBRT (yes vs. no/unknown, HR=1.51, 95% CI 1.26–1.80, P<0.001) and chemotherapy (yes vs. no/unknown, HR=1.79, 95% CI 1.40–2.29, P<0.001) were statistically correlated with decreased OS, interpretations should be done with caution, as addressed in the discussion. In the univariate Cox analysis, the same statistical prognostic factors were estimated as in the multivariate analysis. Detailed Cox statistical analysis related to OS are shown in **Table 2**.

**Table 2:** Univariate and multivariate Cox proportional hazard regression for analyses of medullary thyroid carcinoma patients for overall survival.

	Multivariate			Univariate		
	HR	95% CI	P value	HR	95% CI	P value
Age						
<=45	1	reference		1	reference	
>45 and <=60	1.98	(1.59–2.45)	<0.001	1.97	(1.59–2.45)	<0.001
>60	4.43	(3.62–5.41)	<0.001	4.44	(3.63–5.43)	<0.001
Sex						
Female	1	reference		1	reference	
Male	1.27	(1.11–1.46)	0.001	1.27	(1.11–1.46)	0.001
Race						
White	1	reference		1	reference	
Black	1.37	(1.09–1.73)	0.008	1.37	(1.09–1.73)	0.008
^a^Others	0.73	(0.55–0.98)	0.038	0.74	(0.56–1.00)	0.048
AJCC T stage						
T1				1	reference	
T2				1.37	(0.84–2.22)	0.203
T3				1.03	(0.65–1.62)	0.909
T4				1.30	(0.79–2.16)	0.300
Unspecified				0.87	(0.52–1.45)	0.586
AJCC N stage						
N0	1	reference		1	reference	
N1a	1.03	(0.80–1.32)	0.826	1.05	(0.81–1.37)	0.695
N1b	1.36	(1.07–1.73)	0.013	1.39	(1.08–1.80)	0.012
Unspecified	1.12	(0.84–1.49)	0.435	1.09	(0.80–1.48)	0.595
AJCC M stage						
M0	1	reference		1	reference	
M1	1.98	(1.48–2.65)	<0.001	2.07	(1.52–2.82)	<0.001
Unspecified	1.29	(1.06–1.57)	0.011	1.64	(1.09–2.47)	0.018
Pathological Grade						
Well	1	reference		1	reference	
Moderate	1.06	(0.58–1.95)	0.840	1.04	(0.57–1.90)	0.903
Poor	1.54	(0.91–2.59)	0.104	1.49	(0.88–2.51)	0.136
Undifferentiated	1.76	(0.88–3.54)	0.110	1.71	(0.85–3.44)	0.132
Unspecified	0.78	(0.50–1.23)	0.285	0.77	(0.49–1.22)	0.264
Tumor size						
<=1cm	1	reference		1	reference	
>1cm and <=2cm	1.09	(0.79–1.51)	0.581	1.09	(0.79–1.50)	0.592
>2cm and <=3cm	1.50	(1.09–2.07)	0.013	1.37	(0.97–1.95)	0.076
>3cm and <=4cm	1.77	(1.23–2.53)	0.002	1.59	(1.07–2.37)	0.021
>4cm and <=5cm	1.83	(1.28–2.63)	0.001	1.76	(1.20–2.58)	0.004
>5cm	1.89	(1.34–2.66)	<0.001	1.84	(1.29–2.64)	0.001
Unspecified	1.63	(1.12–2.35)	0.010	1.59	(1.08–2.34)	0.019
Tumor extension						
Confined to thyroid capsule	1	reference		1	reference	
T3b	1.87	(1.43–2.46)	<0.001	1.97	(1.48–2.63)	<0.001
T4a	2.23	(1.67–2.98)	<0.001	2.14	(1.54–2.97)	<0.001
T4b	1.70	(1.20–2.40)	0.003	1.53	(1.01–2.30)	0.042
Unspecified	1.05	(0.78–1.42)	0.752	1.07	(0.77–1.49)	0.691
Surgery						
No surgery	1	reference		1	reference	
Lobectomy	0.29	(0.20–0.40)	<0.001	0.29	(0.20–0.40)	<0.001
Total thyroidectomy	0.25	(0.20–0.30)	<0.001	0.25	(0.20–0.31)	<0.001
Other surgery	0.23	(0.15–0.33)	<0.001	0.23	(0.15–0.33)	<0.001
Neck dissection						
No				1	reference	
Yes				0.93	(0.76–1.14)	0.482
Unspecified				1.01	(0.78–1.31)	0.922
EBRT						
No/Unknown	1	reference		1	reference	
Yes	1.51	(1.26–1.80)	<0.001	1.52	(1.27–1.82)	<0.001
Chemotherapy						
No/Unknown	1	reference		1	reference	
Yes	1.79	(1.40–2.29)	<0.001	1.78	(1.39–2.29)	<0.001
Systemic therapy						
No/Unknown	1	reference		1	reference	
Yes	0.75	(0.62–0.90)	0.002	0.75	(0.62–0.91)	0.004

HR, hazard ratio; CI, confidential interval; ^a^Others, American Indian/Alaska Native, Asian/Pacific Islander; SEER, the Surveillance, Epidemiology, and End Results program; SEER stage: see materials and methods; EBRT, external beam radiation therapy.

**Figure 3:**
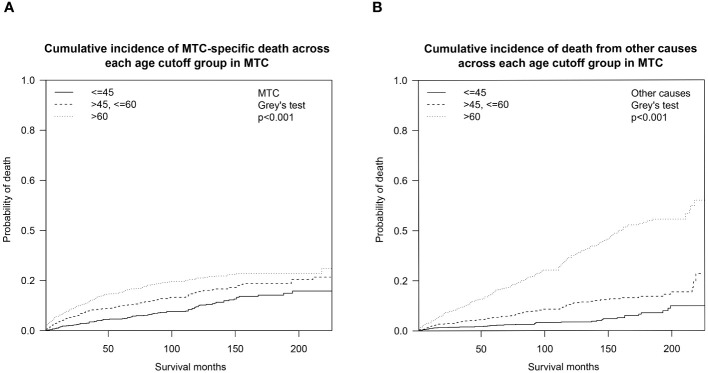
Cumulative incidence curves demonstrating competing risks of death for MTC across age cutoffs set at 45 and 60: **(A)** Cumulative incidence of MTC-specific deaths; **(B)** Cumulative incidence of non-MTC-specific deaths. MTC, medullary thyroid cancer.

We also analyzed the OS by calculating the cumulative incidence of MTC/non-MTC deaths separately in order to further detect the impact of age cutoffs on MTC survival (**Figure 3**). Overall, the cumulative incidences of death from MTC over a 5- and 10-year period were 10.6% (95% CI: 9.5%-11.7%) and 16.0% (95% CI: 14.6%-17.5%), respectively, while the 5- and 10-year cumulative odds of dying from non-MTC causes were 8.5% (95% CI: 7.5%-9.5%) and 15.5% (95% CI: 14.0%-17.0%). Separate 5- and 10-year cumulative incidence of death from MTC causes and non-MTC causes in different age cutoff populations are described in **Table 3**.

**Table 3:** Competing risk hazard regression analysis of survival for medullary thyroid carcinoma patients.

	Death from MTC			Death from OC		
	SHR	95% CI	P value	SHR	95% CI	P value
Age						
<=45	1	reference		1	reference	
>45 and <=60	1.90	(1.37–2.63)	<0.001	2.71	(1.65–4.47)	<0.001
>60	2.45	(1.79–3.35)	<0.001	12.00	(7.52–19.14)	<0.001
Sex						
Female	1	reference		1	reference	
Male	1.30	(1.05–1.61)	0.016	1.17	(0.91–1.50)	0.231
AJCC T stage						
T1	1	reference		1	reference	
T2	0.82	(0.26–2.58)	0.738	1.45	(0.64–3.30)	0.371
T3	0.73	(0.25–2.17)	0.571	1.03	(0.44–2.38)	0.952
T4	1.87	(0.62–5.61)	0.265	0.91	(0.28–2.99)	0.876
Unspecified	0.82	(0.24–2.74)	0.743	3.35	(1.34–8.38)	0.010
AJCC N stage						
N0	1	reference		1	reference	
N1a	2.57	(1.63–4.05)	<0.001	0.53	(0.31–0.93)	0.028
N1b	2.46	(1.57–3.84)	<0.001	1.04	(0.64–1.68)	0.884
Unspecified	2.13	(1.28–3.56)	0.004	0.50	(0.25–1.02)	0.058
AJCC M stage						
M0	1	reference		1	reference	
M1	3.24	(2.00–5.25)	<0.001	1.92	(0.94–3.93)	0.072
Unspecified	2.08	(0.99–4.33)	0.052	1.20	(0.56–2.54)	0.640
Tumor size						
<=1cm	1	reference		1	reference	
>1cm and <=2cm	1.40	(0.67–2.92)	0.370	1.28	(0.78–2.08)	0.330
>2cm and <=3cm	2.36	(1.14–4.88)	0.020	0.90	(0.51–1.60)	0.725
>3cm and <=4cm	2.99	(1.42–6.30)	0.004	0.94	(0.46–1.90)	0.856
>4cm and <=5cm	2.39	(1.12–5.08)	0.024	1.62	(0.81–3.25)	0.175
>5cm	3.05	(1.48–6.27)	0.003	0.97	(0.47–2.00)	0.928
Unspecified	2.81	(1.29–6.10)	0.009	0.79	(0.38–1.62)	0.514
Tumor extension						
Confined to thyroid capsule	1	reference		1	reference	
T3b	2.21	(1.43–3.43)	<0.001	1.02	(0.52–1.99)	0.954
T4a	1.70	(1.04–2.77)	0.034	1.59	(0.69–3.69)	0.276
T4b	1.59	(0.94–2.71)	0.085	0.48	(0.17–1.31)	0.151
Unspecified	0.43	(0.25–0.74)	0.002	0.68	(0.33–1.44)	0.315
Surgery						
No surgery	1	reference		1	reference	
Lobectomy	0.31	(0.17–0.58)	<0.001	0.97	(0.53–1.76)	0.910
Total thyroidectomy	0.34	(0.24–0.48)	<0.001	0.80	(0.51–1.24)	0.312
Other surgery	0.30	(0.16–0.58)	<0.001	0.39	(0.17–0.89)	0.024
Neck dissection						
No	1	reference		1	reference	
Yes	1.05	(0.71–1.54)	0.815	0.71	(0.49–1.02)	0.065
Unspecified	2.28	(1.48–3.53)	<0.001	5.15	(3.23–8.22)	<0.001
EBRT						
No/Unknown	1	reference		1	reference	
Yes	2.04	(1.59–2.60)	<0.001	0.86	(0.57–1.30)	0.474
Chemotherapy						
No/Unknown	1	reference		1	reference	
Yes	2.95	(2.12–4.12)	<0.001	0.64	(0.33–1.22)	0.174
Systemic therapy						
No/Unknown	1	reference		1	reference	
Yes	0.54	(0.39–0.75)	<0.001	0.52	(0.36–0.74)	<0.001

MTC, medullary thyroid carcinoma; OC, other causes; SHR, sub-distribution hazard ratio; CI, confidential interval SEER, the Surveillance, Epidemiology, and End Results program; SEER stage: see materials and methods; EBRT, external beam radiation therapy.

Results of the competing risk hazards regression analysis were estimated and shown in **Table 4**. Advanced age category (e.g. >45 and <=60 vs. <=45: MTC-specific SHR=1.90, 95% CI 1.37–2.63, P<0.001; non-MTC SHR=2.71, 95% CI 1.65–4.47, P<0.001), unknown surgery performed (vs. no surgery: MTC-specific SHR=0.30, 95% CI 0.16–0.58, P<0.001; non-MTC SHR=0.39, 95% CI 0.17–0.89, P=0.024), and lymph node dissection unspecified (vs. lymph node dissection not performed: MTC-specific SHR=2.28, 95% CI 1.48–3.53, P<0.001; non-MTC SHR=5.15, 95% CI 3.23–8.22, P<0.001) were the only factors that are associated with both a greater probability of death from other causes and death from MTC. The only variable that can reduce both MTC-specific and non-MTC death probability was systemic therapy (vs. systemic therapy not given or unknown: MTC-specific SHR=0.54, 95% CI 0.39–0.75, P<0.001; non-MTC SHR=0.52, 95% CI 0.36–0.74, P<0.001), which can be considered to be a combination of surgery and targeted therapy in the context of MTC treatment. A total thyroidectomy was the only way to reduce death risks from both MTC (vs. no surgery, SHR=0.42, 95% CI 0.25–0.71, P=0.001) and non-MTC causes (vs. no surgery, SHR=0.60, 95% CI 0.25–0.71, P=0.001). Male gender (vs. female: MTC-specific SHR=1.30, 95% CI 1.05–1.61, P=0.016), larger tumor size (e.g., >2cm and <=3cm vs. <=1cm: MTC-specific SHR=2.36, 95% CI 1.14–4.88, P=0.020), more advanced tumor extension (e.g., T3b vs. confined to thyroid capsule: MTC-specific SHR=2.21, 95% CI 1.43–3.43, P<0.001), higher N stage (e.g., N1b vs. N0: MTC-specific SHR=2.46, 95% CI 1.57–3.84, P<0.001), and higher M stage (e.g., M1 vs. M0: MTC-specific SHR=3.24, 95% CI 2.00–5.25, P<0.001) were solely correlated with worse MTC-specific mortality. In addition, surgery (e.g., total thyroidectomy vs. no surgery: MTC-specific SHR=0.34, 95% CI 0.24–0.48, P<0.001) was associated with decreased risk of MTC-specific death. External beam radiation therapy (EBRT vs. no EBRT: MTC-specific SHR=2.04, 95% CI 1.59–2.60, P<0.001) and chemotherapy (vs. no chemotherapy: MTC-specific SHR=2.95, 95% CI 2.12–4.12, P<0.001) seemed to correlate with an increased CSS death rate, but this is open to interpretation.

**Table 4:** Cumulative incidences of death of MTC patients across age cutoffs.

	5-year death risk (95% CI)		10-year death risk (95% CI)	
	MTC (%)	OC (%)	MTC (%)	OC (%)
**Overall**	10.6 (9.5–11.7)	8.5 (7.5–9.5)	16.0 (14.6–17.5)	15.5 (14.0–17.0)
Age cutoffs				
<=45	4.8 (3.5–6.3)	2.1 (1.3–3.2)	10.0 (7.8–12.5)	3.5 (2.3–5.0)
>45 and <=60	10.0 (8.2–11.9)	5.4 (4.1–6.9)	15.6 (13.1–18.2)	10.9 (8.7–13.4)
>60	15.6 (13.5–17.7)	16.2 (14.1–18.5)	21.0 (18.5–23.7)	30.1 (26.8–33.4)

CI, confidential interval; MTC, medullary thyroid carcinoma; OC, other causes.

## Discussion

The prognosis of sporadic MTC patients depends on many known factors. Within the limited sample size of MTC patients, it has been reported that older age, higher-grade lesions, more advanced stage, longer calcitonin doubling time, and incomplete surgical resection convey worse survival outcomes (2, 19, 25–27). With exception, our investigations suggested that age at diagnosis and TNM stage were independent predictors of survival. The staging system of a malignancy was supposed to be the most dominant independent survival predictor by definition. However, the AJCC staging system of MTC failed to present a discriminant survival disparity [5-year OS: 95% in stage I, 91% in stage II, and 89% in stage III; 5-year CSS: 100% in stage I, 99% in stage II, and 97% in stage III (16)]. In light of these rational facts, an optimal age cutoff may be incorporated into developing a more reasonable MTC staging method. Our data offers evidence to guide attempts to improve the staging of MTC.

As the first step to assessing how age impacts OS probability, we fitted a smooth curve using a generalized additive model (GAM) in order to examine whether the relationship between age and overall death risk is linear or there is a threshold effect. **Figure 1** clearly illustrated that the association between age at diagnosis and overall relative risk of death (Log RR) was linear (Likelihood ratio test, P<0.001; Wald test, P<0.001; log rank test, P<0.001). As a result of this discovery, we speculate that cumulative death risks should be smoothly partitioned into equal intervals based on certain age cutoffs. To illustrate this, we plotted five K-M survival curves (**Figure 2A**), representing age groups with cutoffs set at 45, 50, 55, and 60 years. Overlaps were observed between K-M curves representing patients aged 45–50, 50–55, and 55–60, which indicates that survival differences for these age subgroups are not statistically significant. Thus, it is statistically justified to merge all groups from age 45 through 60 years. This is shown in contrast to another set of K-M curves in **Figure 2B**. Accordingly, the study cohort was divided into 3 subgroups with 2 age cutoffs set at 45 and 60 years. In the competing risk analysis, we further calculated 5-year and 10-year cumulative death risks across the 3 age cutoff groups by 2 mutually exclusive causes of death (MTC-specific death and non-MTC-specific death). According to **Table 3**, each older age cutoff population has resulted in a roughly “5%” constant increase in MTC-specific death risks (5-year CSS comparison among <=45, >45 and <=60, >60 subgroups: 4.8% vs. 10.0% vs. 15.6%; 10-year CSS comparison among <=45, >45 and <=60, >60 subgroups: 10.0% vs. 15.6% vs. 21.0%) and an approximately “3 times” exponential increase in non-MTC-specific death risks (5-year non-CSS comparison among <=45, >45 and <=60, >60 subgroups: 2.1% vs. 5.4% vs. 16.2%; 10-year non-CSS comparison among <=45, >45 and <=60, >60 subgroups: 3.5% vs. 10.9% vs. 30.1%). Consequently, for MTC patients, age cutoffs set at 45 and 60 years yield a discriminant survival disparity.

Our results are generally in agreement with previously published articles showing an association indicating that advanced age has a detrimental effect on OS/CSS among MTC patients (28–31). Zeyad et al. have also reported that increased age is an independent predictor of CSS in patients with MTC (19). The study cohort was stratified by a different age (18–64, 65–79, and 80 years) subgroup aiming to make comparisons across varied ages in adults. Their main conclusion was statistically in agreement with ours, but the K-M curves for age subgroups did not provide an ideal way of discriminating survival rates. Another retrospective study led by Shekhar et al. focusing on MTC patients older than 45 years concluded that increasing age and advanced stage of presentation were associated with worse survival with HR 1.05 (p < 0.001) and HR 3.68 (p < 0.001), respectively (18). Patients were subdivided into 3 age groups (45–64, 65–84, and >=84) and demonstrated significant differences in survival times. However, survival curves were not provided in the main text. The most recent SEER database analysis focusing on stage I MTC patients reported (32) that the death risk of MTC patients rose sharply with increases in age beyond 60 years, which is consistent with our smooth curve fitting shown in **Figure 1**. However, the study included the stage I MTC population only and merely compared survival disparities between patients older and younger than 60 years.

We have also determined some other independent adverse outcome predictors listed by impact: older age; higher AJCC M stage; more invasive tumor extension; larger tumor size; more advanced AJCC N stage; black ethnicity; and male gender. All of these predictors were in agreement with previously reported findings (28, 29, 31). This order of predictor’s impact on survival prognosis was another piece of evidence supporting the conclusion that age should be included in forming a more comprehensive staging method for MTC.

As one can expect, surgery and systemic therapy were found to be the only factors that improved survival outcomes of MTC patients. On the contrary, EBRT and chemotherapy, which are well-known as effective therapeutic approaches for many malignancies (33), may have played a deleterious role in MTC treatment (see **Tables 2**, **4**). This was in agreement with an analysis of the SEER data from 1998 to 2004 (34), indicating that adjuvant EBRT showed no overall survival benefit in patients with MTC and positive lymph nodes. The 2015 revised ATA management for MTC (2) recommended that the potential benefits must be weighed against the acute and chronic toxicity associated with the therapy (grade C recommendation). Traditional chemotherapy for MTC management has not been mentioned in commonly used guidelines (2, 35, 36). Alternatively, the analogy for the association between EBRT/chemotherapy and MTC treatment could be interpreted as “fighting a losing battle.” That is, MTC patients who were treated with adjuvant EBRT or chemotherapy were people with late-stage disease with complicated recurrences or distant metastases, and were destined for short survival with or without treatment of any kind.

Our study has several highlights. Firstly, to our knowledge, this study represents the largest and most updated cohort of patients with pathologically confirmed MTC. Secondly, our study demonstrates in detail the precise relationship between age and survival outcome, and the process of defining the age cutoffs. Moreover, public databases such as SEER provide more generalizable and representative data than single centers. Lastly, the competing risk model considers other competing events, providing a deeper level of understanding of the impact of age on differential survival.

Nevertheless, there are also several limitations to the current study. We acknowledge that the MTC patient population is genetically diverse, with approximately 75% of cases being sporadic and the remaining 25% associated with RET mutations. These familial cases, which are linked to multiple endocrine neoplasia type 2 (MEN2), potentially exhibit unique survival outcomes due to access to RET-targeted therapies and the specific pathophysiology of MEN2. However, RET mutation status, CEA, calcitonin levels, and doubling time were not recorded in the SEER database. Besides, the “systemic therapy” described in the database did not provide sufficient details about the targeting regimen and the sequence of surgery and targeted therapy.

Despite these limitations, we have conducted a large-scale SEER-based study using a competing risk model to evaluate the impact of age on survival outcomes of MTC patients. A future prospective, multi-center study is recommended to validate these findings, which could contribute to the development of a more comprehensive staging system for Medullary Thyroid Carcinoma (MTC).

## Data availability statement

The datasets presented in this study can be found in online repositories. The names of the repository/repositories and accession number(s) can be found below: https://seer.cancer.gov/.

## Ethics statement

Ethical approval was not required for the study involving humans in accordance with the local legislation and institutional requirements. Written informed consent to participate in this study was not required from the participants or the participants’ legal guardians/next of kin in accordance with the national legislation and the institutional requirements.

## Author contributions

KZ: Conceptualization, Formal analysis, Investigation, Methodology, Software, Validation, Visualization, Writing – original draft, Writing – review & editing. XW: Formal analysis, Writing – original draft. TW: Supervision, Writing – review & editing. ZL: Conceptualization, Supervision, Writing – review & editing. JZ: Writing – review & editing. Y-WC: Conceptualization, Data curation, Supervision, Validation, Writing – review & editing.
